# Larval Performance and Kill Rate of Convergent Ladybird Beetles, *Hippodamia convergens*, on Black Bean Aphids, *Aphis fabae*, and Pea Aphids, *Acyrthosiphon pisum*

**DOI:** 10.1673/031.013.4601

**Published:** 2013-05-31

**Authors:** Travis M. Hinkelman, Brigitte Tenhumberg

**Affiliations:** 1 School of Biological Sciences, University of Nebraska, Lincoln, NE 68588, USA; 2 Department of Mathematics, University of Nebraska, Lincoln, NE 68588, USA

**Keywords:** Aphididae, Coleoptera, Coccinellidae, generalist predator, Hemiptera, toxic prey

## Abstract

Generalist predator guilds play a prominent role in structuring insect communities and can contribute to limiting population sizes of insect pest species. A consequence of dietary breadth, particularly in predatory insects, is the inclusion of low-quality, or even toxic, prey items in the predator's diet. Consumption of low-quality prey items reduces growth, development, and survival of predator larvae, thereby reducing the population sizes of generalist predators. The objective of this paper was to examine the effect of a suspected low-quality aphid species, *Aphis fabae* (Scopoli) (Hemiptera: Aphididae), on the larval performance of an abundant North American predator, *Hippodamia convergens* (Guérin-Méneville) (Coleoptera: Coccinellidae). For comparison, *H. convergens* larvae were also reared on a known high-quality aphid species *Acyrthosiphon pisum* (Harris) (Hemiptera: Aphididae) and on a 50:50 mix of both aphid species. The proportion of *H. convergens* larvae surviving to the adult stage was dramatically lower (0.13) on the *A. fabae* diet than on the *A. pisum* diet (0.70); survival on the mixed diet was intermediate (0.45) to survival on the single-species diets. Similarly, surviving *H. convergens* larvae also developed more slowly and weighed less as adults on the *A. fabae* diet than on the *A. pisum* diet. Despite the relatively poor performance on the *A. fabae* diet, *H. convergens* larvae killed large numbers of *A. fabae*. Furthermore, *H. convergens* displayed a preference for *A. fabae* in the mixed diet treatment, most likely because *A. fabae* was easier to catch than *A. pisum*. The results suggest that increases in the distribution and abundance of *A. fabae* in North America may have negative effects on *H. convergens* population size.

## Introduction

Generalist predators play a prominent role in structuring insect communities through intraguild predation (Rosenheim et al. 1995), apparent competition (van Veen et al. 2006), and tritrophic interactions (Evans 2008). The numerous potential interactions that involve generalist predators complicate predictions about when generalist predator guilds can contribute to limiting insect pest populations (Obrycki et al. 2009; Weber and Lundgren 2009), which has produced a contentious debate about the overall effectiveness of generalist predators in biological control (Kindlmann and Dixon 2001; Symondson et al. 2002). One factor able to reduce the effectiveness of top-down control by generalist predators is the presence of non-target prey (Harmon and Andow 2004; Koss and Snyder 2005; Prasad and Snyder 2006), particularly if the non-target prey species is toxic (van Veen et al. 2009), more frequently encountered (Bergeson and Messina 1998), or easier to capture (Provost et al. 2006) than the target prey species. In this study, the costs of consuming a suspected low-quality prey species were measured on a generalist predator both in the presence and absence of a known high-quality prey species.

Consumption of toxic prey is particularly likely when high-quality prey are scarce because generalist predators respond to the threat of starvation by including low-quality and toxic prey items in their diet (Dixon 2000; Sloggett and Majerus 2000; Sherratt et al. 2004). Even when high-quality prey are abundant, the availability of high-quality prey to predators may be low if the prey are difficult to catch and subdue (Lang and Gsödl 2001; Provost et al. 2006). Generally, there is a trade-off between chemical defense and alternative defense mechanisms (Pasteels 1983), suggesting that predators can capture toxic prey more easily than high-quality prey. As a consequence, the vulnerability of prey to pre dation often plays a more prominent role in predators' diet selection than the nutritional quality or toxicity of prey (Sih and Christensen 2001).

**Table 1.**
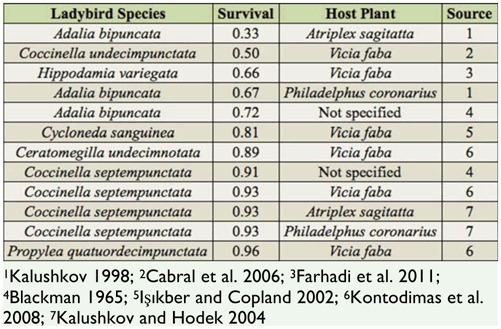
Proportion of individuals surviving to the adult stage for several species of ladybird beetle larvae when reared on a diet of *Aphis fabae*.

*Aphis fabae* (Scopoli) (Hemiptera: Aphididae) is a polyphagous cosmopolitan pest (Dixon 1998) and varies widely in quality as food (Table 1) for aphidophagous ladybird beetles (Coleoptera: Coccinellidae), which are prominent generalist predators in insect communities (Obrycki and Kring 1998; Obrycki et al. 2009; Weber and Lundgren 2009). However, the quality of *A. fabae* as a food for one of the most abundant native ladybird beetles in North America, *Hippodamia convergens* (Guérin-Méneville), is unknown. *A. fabae* was introduced to North America from Europe about 130 years ago and has achieved pest status (Foottit et al. 2006). Moreover, *A. fabae* may become more prevalent in North America, because global climate change is expected to increase yields of grain legumes, which include important host plants for *A. fabae* such as broad beans, *Vicia faba* (L.) (Fabales: Fabaceae) (Andrews and Hodge 2010). In general, ladybird beetles often show no preference for high-quality prey and even consume toxic prey in laboratory studies (Blackman 1967a; Nielsen et al. 2002; Ferrer et al. 2008; Nedvěd and Salvucci 2008). Thus, if *A. fabae* is a low-quality food for *H. convergens*, consumption of *A. fabae* may have negative effects on *H. convergens* populations, which could cascade through the insect community and potentially impact the strength of top down control imposed by *H. convergens* on aphid pests.

The central objective of this study was to measure the larval performance of *H. convergens* on a diet of *A. fabae*. For comparison, larval performance was also measured for *H. convergens* on a diet of *Acyrthosiphon pisum* (Harris) (Hemiptera: Aphididae), which is a high-quality food for a large number of coccinellid species (Rana et al. 2002; Ueno 2003; Kalushkov and Hodek 2004), including *H. convergens* (Giles et al. 2001). Like *A. fabae, A. pisum* was introduced to North America from Europe about 130 years ago and has achieved pest status (Foottit et al. 2006). *A. pisum* and *A. fabae* both exploit *V. faba* and *Pisum sativum* (L.) (Fabales: Fabaceae) as host plants (van Emden and Harrington 2007). Moreover, *A. pisum* readily colonizes *V. faba* plants containing *A. fabae* in the laboratory (Hinkelman and Tenhumberg, unpublished data). The presence of multiple prey species on a plant (or in a field) can alter the topdown effects of a generalist predator via changes in predator preferences and performance (Harmon and Andow 2004; Evans 2008). Thus, prey preference and performance of *H. convergens* were examined on a diet comprised of both aphid species. Laboratory tests of prey preferences provide a baseline test of the potential negative effects of toxic prey on generalist predators.

## Materials and Methods

*A. pisum* and *A. fabae* were maintained in separate cultures with *V. faba* as the host plant. Adult *H. convergens* were housed in cages with *A. pisum* and *V. faba*. Adult *H. convergens* were purchased from a commercial supplier (A-1 Unique Insect Control, www.a-lunique.com), who collects *H. convergens* from the Sierra Nevada Mountains and maintains them in dormant state through cold storage (3° C). All insects were maintained at approximately 24° C on a 16:8 L:D photoperiod. To avoid egg cannibalism, eggs were removed from the *H. convergens* culture and placed in a separate cage for hatching. Recently hatched (< 24 hrs) *H. convergens* larvae were placed individually in plastic vials (diameter = 26 mm; height = 67 mm; volume = 33 mL) and randomly assigned to one of three diet treatments: (1) *A. fabae* only, (2) *A. pisum* only, and (3) 50:50 mix of *A. fabae* and *A. pisum*. Neonate larvae were not weighed at the start of the experiment, but random treatment assignments, and a relatively large sample size, made it unlikely that a systematic bias in initial condition was introduced into the experimental design.

**Figure 1.**
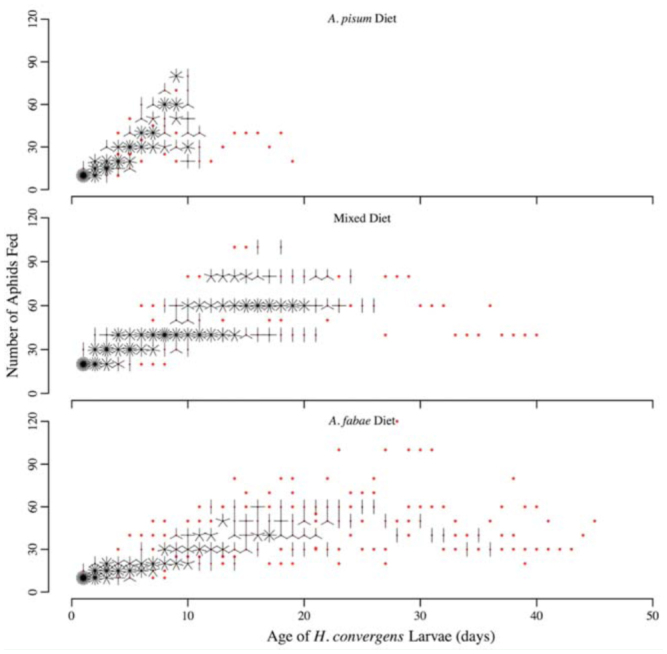
Sunflower plots of number of aphids fed each day to *Hippodamia convergens* larvae on three diet treatments: *Aphis fabae* alone, *Acyrthosiphon pisum* alone, and 50:50 mix of *A. fabae* and *A. pisum*. The number of ‘petals’ on the sunflower indicates the number of *H. convergens* larvae fed that number of aphids at that age; red points indicate a single larva. High quality figures are available online.

Each day, the live and dead aphids remaining in each predator's vial were counted and removed. Dead aphids were divided into two categories: those that showed evidence of piercing by the mouthparts of *H. convergens* larvae (killed) and those with no evidence of piercing (dead). The number of aphids killed each day was determined by subtracting the number of live and dead aphids from the number of aphids supplied the previous day. *H. convergens* larvae were provided with fresh aphids daily. The number of aphids fed each day (Figure 1) was based on the number of aphids killed on the previous day. Thus, feeding was tailored to each individual *H*. *convergens* larvae and did not follow a set schedule. Across all three treatments, aphids were subjectively size-matched by selecting large *A. fabae* and similarly-sized *A. pisum* to ensure that differences in preference or performance were not attributable to aphid size differences, because apterous *A. pisum* adults (3.8 mg) are 4× larger than apterous *A. fabae* adults (0.9 mg) (Dixon and Kindlmann 1999).

Three measures of *H. convergens* performance were examined: (1) survival to the adult stage (binary response), (2) time to adult stage (days), and (3) adult mass (mg). Adult fecundity was not measured, because fecundity is typically highly variable for predatory insects and thus requires a large sample size to obtain a good estimate. A sufficiently large sample size was difficult to get because of the low survival rate on the *A. fabae* diet. However, adult size is positively correlated with reproductive capacity (Stewart et al. 1991), thus adult weight was used as an indicator of *H. convergens* fitness. The relationship between diet treatment and performance variables was analyzed with either a generalized linear model with a binomial error distribution (survival) or linear models with normal error distributions (developmental time, mass). The overall effect of the diet treatment on each performance variable was tested with either analysis of deviance (survival) or analysis of variance (developmental time, mass).

Locally weighted polynomial regression models were fit separately for each diet treatment to characterize the relationship between the number of aphids killed each day and the age of *H. convergens* larvae. The data were split into two subsets based on whether or not *H. convergens* larvae survived to the adult stage, because the number of aphids killed at a given age was related to the developmental stage of the larvae, and unsuccessful larvae typically developed more slowly than successful larvae.

**Table 2.**
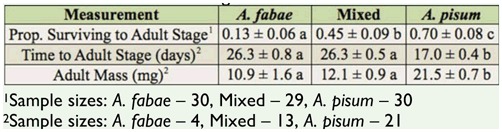
Performance of *Hippodamia convergens* larvae on three diet treatments: *Aphis fabae* alone, *Acyrthosiphon pisum* alone, and 50:50 mix of *A. fabae* and *A. pisum*. Values presented are the predicted means ± standard error from the statistical models. Values followed by different letters are significantly different. Estimates for developmental time and mass include only larvae that survived to the adult stage.

For *H. convergens* larvae on the mixed diet, prey preferences were tested with a two-tailed sign test by comparing the total number of each aphid species killed over the duration of the larval period. A significant prey preference, therefore, indicates that the two aphid species were not killed in the same proportion as available in the environment (Sih and Christensen 2001). R was used to conduct all statistical analyses (R Development Core Team 2011).

## Results

**Figure 2.**
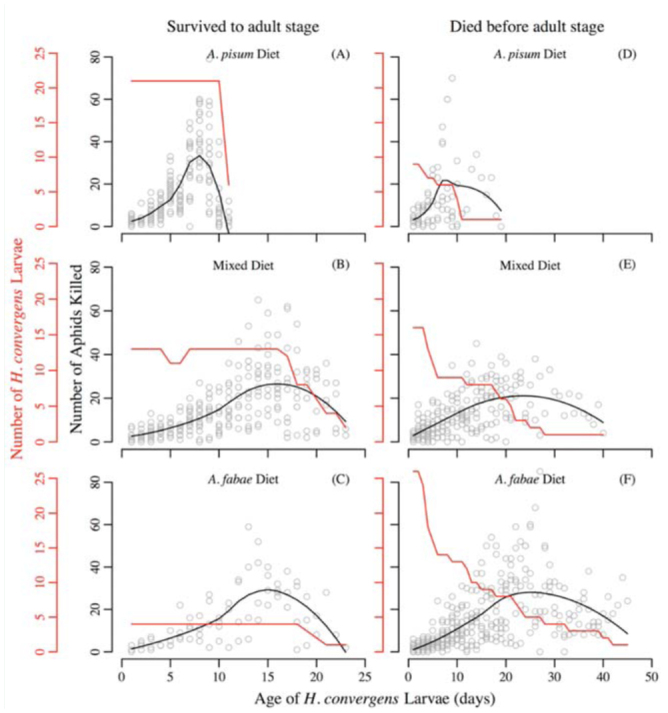
Number of aphids killed each day by *Hippodamia convergens* larvae on three diet treatments: *Aphis fabae* alone, *Acyrthosiphon pisum* alone, and 50:50 mix of *A. fabae* and *A. pisum*. Black lines are locally weighted polynomial regression models of aphids killed each day. Data was divided based on the fate of the *H. convergens* larvae. [Note the different range of the x-axis for (A–C) and (D–F).] Red lines indicate the number of *H. convergens* larvae receiving food each day. The early dip in the red line in (B) arises from missing data because of a data recording error rather than through pupation or death of *H. convergens* larvae. High quality figures are available online.

**Figure 3.**
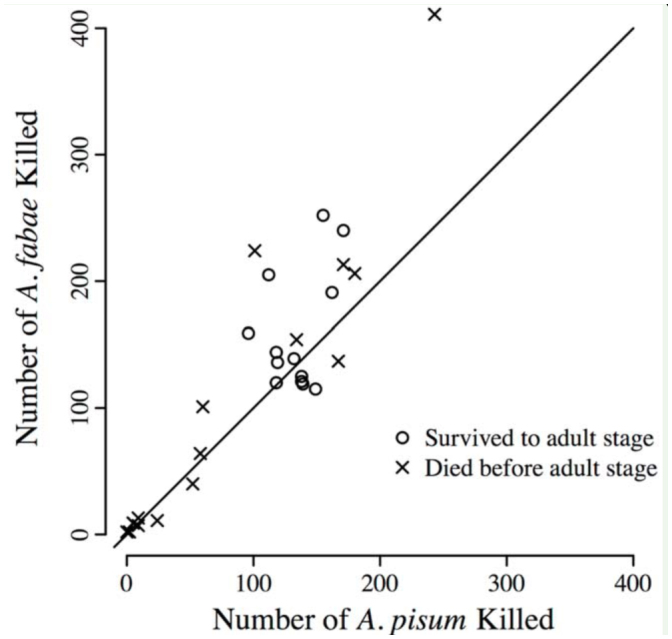
Total number of aphids killed over the duration of the larval period for *Hippodamia convergens* on a 50:50 mix diet *of Acyrthosiphon pisum* and *Aphis fabae*. Symbols indicate fate of *H. convergens* larvae. The large variation in the number of aphids killed reflects variation in the number of days *H. convergens* spent in the larval stage before pupating or dying (see Figure 2B, E). Reference line indicates no difference in number of *A. pisum* and *A. fabae* killed. High quality figures are available online.

Diet treatment significantly affected all three performance measures (survival to the adult stage: *deviance*_2,86_ = 21.4, *p* < 0.001; time to the adult stage: *F*2,_35_ = 139.9, *p* < 0.001; adult mass: *F*_2,35_ = 45.9, *p* < 0.001). Survival was significantly higher on a diet comprised of *A. pisum* (0.70) than *A. fabae* (0.13); survival on the mixed diet (0.45) was intermediate to survival on the diets of single aphid species (Table 2). Developmental time to the adult stage was significantly shorter, and adult mass was significantly greater, on the *A. pisum* diet than on either of the other two diets (Table 2). The number of aphids killed by *H. convergens* larvae peaked earlier on the *A. pisum* diet (8 days; Figure 2A) than on either the mixed (16 days; Figure 2B) or *A. fabae* diets (15 days; Figure 2C). Although *H. convergens* larvae performed better when fed *A. pisum*, larvae on the mixed diet killed significantly fewer *A. pisum* than *A. fabae* over the duration of the larval period (sign test, *p* = 0.024; Figure 3).

## Discussion

The objective of this study was to examine the fitness consequences of consuming the insect pest *A. fabae* on a native predatory insect in North America, namely *H. convergens*. The results suggest that *A. fabae* is a very low-quality prey that drastically influences three measures of *H. convergens* performance. An *A. fabae* diet increases developmental time and reduces survival and adult mass of *H. convergens* larvae relative to the high-quality aphid *A. pisum*. Consuming *A. fabae* increased the developmental time of *H. convergens* larvae, resulting in a delay in peak killing capacity relative to the *A. pisum* diet. The predator larvae took a very long time to pupate or die on the *A. fabae* diet (Figure 2C, F) and, as a consequence, they killed as many aphids on the *A. fabae* (202 ± 37 aphids/larva) diet as larvae on the *A. pisum* diet (148 ± 31 aphids/larva) over their entire larval periods (generalized linear model: *t* = -1.12, df = 58, *p* = 0.27). The findings are not limited to *A. fabae* grown on *V. faba;* using sugar beets, *Beta vulgaris*, as a host plant produced a similarly negative effect for *H. convergens* larvae (Tenhumberg, unpublished data). To our knowledge, larval survival on an *A. fabae* diet is lower for *H. convergens* than any other ladybird beetle species previously tested (Table 1). Although compounds sequestered from host plants can contribute to aphid defense (Pasteels 2007), there is no clear effect of host plant on suitability of *A. fabae* for ladybird beetles (Table 1).

The poor performance on diets that included *A. fabae* in this study was unlikely to have been caused by prey limitation, because excess aphids were provided daily, and *H. convergens* rarely fully consume *A. fabae* individuals (Hinkelman 2012). Partial consumption of *A. fabae* has also been reported for *Adalia bipunctata* (Blackman 1967b). Furthermore, behavioral experiments show that *H. convergens* larvae spend nearly 9× longer handling *A. fabae* than size-matched *A. pisum* (Hinkelman 2012), suggesting that *H. convergens* may be limited by time rather than aphid abundance on the *A. fabae* diet.

Interestingly, *H. convergens* larvae readily consumed *A. fabae* (either partially or fully) even if *A. pisum* was available in excess. Moreover, *H. convergens* exhibited a significant preference for *A. fabae* on the mixed diet despite the negative effects of *A. fabae* on larval performance. This ostensibly suboptimal foraging behavior might have been the result of effective anti-predator behavior by *A. pisum* (Francke et al. 2008) that reduced the capture success of *H. convergens* larvae even in the relatively simple environment of a plastic tube (i.e., by dropping from sides and lid). Indeed, *A. pisum* is less vulnerable to predation by *H. convergens* adults than *A. fabae* in laboratory tests on alfalfa plants (Bernays 1989). Our results are consistent with the growing appreciation that predatory insects commonly select prey for factors (e.g., mobility) other than nutritional value (Eubanks and Denno 2000; Sih and Christensen 2001). The relative vulnerability of *A. pisum* and *A. fabae* is also likely affected by aphid age. Young aphids are generally less mobile (Tokunaga and Suzuki 2007) and less likely to drop from plants (Losey and Denno 1998; Gish et al. 2012) than adult aphids. Thus, the age distribution of *A. pisum* and *A. fabae* populations is likely to affect the diet composition of *H. convergens* larvae in the field. It is not known if the quality of *A. fabae* depends on aphid age, but *H. convergens* larvae also performed poor-poorly on a diet comprised of a random mix of *A. fabae* instars relative to a random mix of *A. pisum* instars (Tenhumberg, unpublished data).

These experiments were conducted in an artificial laboratory setting lacking foraging cues (e.g., honeydew) and behaviors (e.g., oviposition) that are present in the field. Aphid honeydew is used as a foraging cue in some aphid-coccinellid systems (Carter and Dixon 1984; Ide et al. 2007), but *H. convergens* larvae do not discriminate between *A. fabae* and *A. pisum* based on aphid honeydew (Purandare and Tenhumberg 2012). It is possible that adult ladybird beetles avoid ovipositing on plants infested with *A. fabae* in the field. However, it is largely unknown whether ladybird beetles preferentially oviposit near highquality aphid species (Omkar and Mishra 2005; Fréchette et al. 2006). Moreover, fields, and even individual plants, are likely to contain more than one prey species, which complicates the oviposition decisions of generalist predatory insects. More work is needed to determine the extent to which ladybird beetles use behavioral mechanisms to avoid consuming low quality and toxic prey.

Caution is required when extrapolating the results of laboratory studies to field conditions. In the field, predator and prey rarely interact on a strictly one-to-one basis, and the numerous indirect interactions associated with multispecies communities complicate biological control predictions (Müller and Godfray 1999; Harmon and Andow 2004). For example, generalist predators can mediate positive, negative, or neutral indirect interactions between prey species (Harmon and Andow 2004; Evans 2008). A recent study in a syrphid-aphid system (Diptera: Syrphidae) provides a particularly interesting parallel to our study system (van Veen et al. 2009). In that study, a positive indirect effect of a low-quality prey species on a high-quality prey species was proposed to arise from the effect of the low-quality prey species on the shared predator, i.e., low-quality prey slowed development and reduced larval survival of the predator, thereby reducing total prey consumption (van Veen et al. 2009). The poor larval performance of *H. convergens* on an *A. fabae* diet suggests that *A. fabae* might have a positive indirect effect on aphid species that share *H. convergens* as a predator. However, the large number of *A. fabae* individuals killed by *H. convergens* larvae could counteract any positive indirect effects associated with high mortality of *H. convergens* larvae. Understanding the conditions leading to positive indirect interactions among aphid species is a promising area for future research with important implications for biological control. In conclusion, the results of our study suggest that increases in the distribution and abundance of *A. fabae* in North America could have negative effects on *H. convergens* population size, which might have implications for the indirect interactions among aphid species.
